# Insulin-like growth factor 2 hypermethylation in peripheral blood leukocytes and colorectal cancer risk and prognosis: a propensity score analysis

**DOI:** 10.3389/fonc.2023.971435

**Published:** 2023-05-05

**Authors:** HongRu Sun, YanLong Liu, YuXue Zhang, Yibaina Wang, YaShuang Zhao, YuPeng Liu

**Affiliations:** ^1^ Department of Epidemiology, Public Health College, Harbin Medical University, Harbin, Heilongjiang, China; ^2^ Department of Colorectal Surgery, The Third Affiliated Cancer Hospital of Harbin Medical University, Harbin, Heilongjiang, China; ^3^ Department of Hygiene Microbiology, Public Health College, Harbin Medical University, Harbin, Heilongjiang, China

**Keywords:** *IGF2* hypermethylation, colorectal neoplasms, risk, prognosis, propensity score analysis

## Abstract

**Background:**

To comprehensively assess and validate the associations between insulin-like growth factor 2 (*IGF2*) gene methylation in peripheral blood leukocytes (PBLs) and colorectal cancer (CRC) risk and prognosis.

**Methods:**

The association between *IGF2* methylation in PBLs and CRC risk was initially evaluated in a case-control study and then validated in a nested case-control study and a twins’ case-control study, respectively. Meanwhile, an initial CRC patient cohort was used to assess the effect of *IGF2* methylation on CRC prognosis and then the finding was validated in the EPIC-Italy CRC cohort and TCGA datasets. A propensity score (PS) analysis was performed to control for confounders, and extensive sensitivity analyses were performed to assess the robustness of our findings.

**Results:**

PBL *IGF2* hypermethylation was associated with an increased risk of CRC in the initial study (OR_PS-adjusted_, 2.57, 95% CI: 1.65 to 4.03, *P*<0.0001), and this association was validated using two independent external datasets (OR_PS-adjusted_, 2.21, 95% CI: 1.28 to 3.81, *P*=0.0042 and OR_PS-adjusted_, 10.65, 95% CI: 1.26 to 89.71, *P*=0.0295, respectively). CRC patients with *IGF2* hypermethylation in PBLs had significantly improved overall survival compared to those patients with *IGF2* hypomethylation (HR_PS-adjusted_, 0.47, 95% CI: 0.29 to 0.76, *P*=0.0019). The prognostic signature was also observed in the EPIC-Italy CRC cohort, although the HR did not reach statistical significance (HR_PS-adjusted_, 0.69, 95% CI: 0.37 to 1.27, *P*=0.2359).

**Conclusions:**

*IGF2* hypermethylation may serve as a potential blood-based predictive biomarker for the identification of individuals at high risk of developing CRC and for CRC prognosis.

## 1 Introduction

Colorectal cancer (CRC) is the third-most common cancer in men and the second-most in women worldwide, with an estimated 1,931,590 newly diagnosed cases and 935,173 deaths in 2020, accounting for approximately 1 in 10 cancer cases and deaths (1, 2). In China, the number of CRC cases has rapidly increased since the 1980s, with an estimated 555,477 newly diagnosed patients and 286,162 deaths in 2020, accounting for approximately 30% of all annually diagnosed CRC cases and CRC-related deaths worldwide (2, 3). The initiation and progression of CRC is multifactorial and gradual with progressive accumulation of both genetic and epigenetic abnormalities, including aberrant DNA methylation and loss of imprinting (LOI) (4–6). The insulin-like growth factor 2 (IGF2) gene is one of the first imprinted genes identified in humans. The IGF2 protein has a tumour-promoting effect on existing colorectal neoplasia (7–10) and that LOI of IGF2 in either tissue or peripheral blood leukocyte (PBL) samples is associated with an increased risk of CRC (11, 12). Furthermore, a recent cohort study showed a significant association between IGF2 hypomethylation in paraffin-embedded tissues and poor CRC prognosis (13). However, no study has evaluated whether PBL IGF2 methylation, which can be determined using non-invasive techniques, is associated with CRC risk or prognosis.

We therefore performed this study to comprehensively assess the association between PBL IGF2 methylation status and CRC risk and prognosis. The propensity score (PS) method has been increasingly used to reduce the likelihood of confounding bias in observational studies. It is a powerful statistical tool to control for confounding bias and is often more practical and statistically more efficient than conventional strategies including covariate matching, stratified analysis, and multivariate statistical analysis (14, 15). In this study, we used PS-based methods to assess the effect of PBL IGF2 methylation on the risk of developing CRC, and then further validated our findings using external datasets from EPIC-Italy CRC cohort and GEO (16–18). In addition, we used the same PS-based methods to assess the association between IGF2 methylation and CRC prognosis using CRC patient PBLs and tumour tissues, and then further validated our findings using external datasets from EPIC-Italy CRC cohort and The Cancer Genome Atlas (TCGA) (17, 19).

## 2 Materials and methods

### 2.1 Study design and participants

#### 2.1.1 Initial study

##### 2.1.1.1 *IGF2* methylation in PBLs and CRC risk

**Figure 1:**
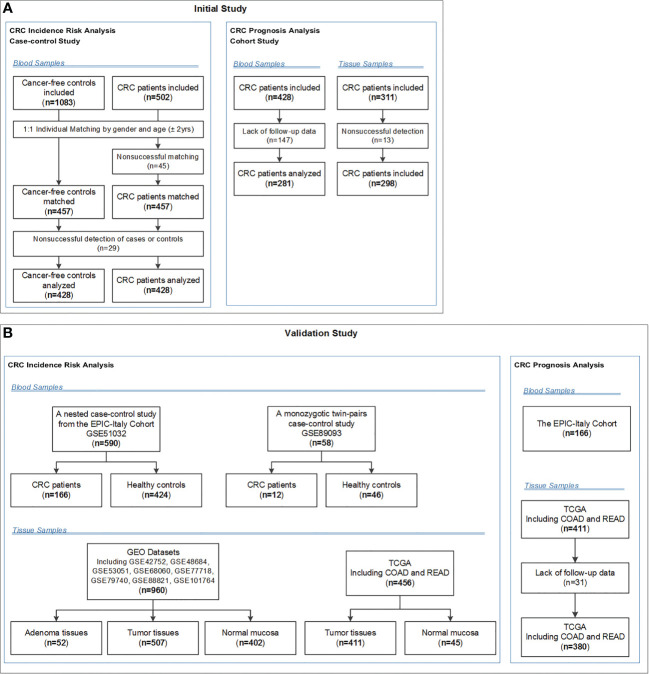
Flow chart of participants included and analysed in the **(A)** initial and **(B)** validation studies. COAD, colon adenocarcinoma; CRC, colorectal cancer; EPIC-Italy, the European Prospective Investigation into Cancer and nutrition (EPIC cohort) in Italy; GEO, Gene Expression Omnibus; READ, rectum adenocarcinoma; TCGA, The Cancer Genome Atlas.

Descriptions of the study design and patient selection strategy have been published elsewhere (20, 21). **Figure 1** showed the selection strategy of the subjects. We included 1083 primary sporadic CRC cases diagnosed at the Third Affiliated Hospital (from June 2004 to May 2005 and May 2007 to January 2008) and the Second Affiliated Hospital (from October 2010 to December 2011) of Harbin Medical University (HMU) in Harbin, China. A total of 502 Cancer-free controls were selected contemporaneously from the Second Affiliated Hospital of HMU. Then controls were individually matched to each case (1:1) according to gender and age ( ± 2 years). Because no eligible matched controls were found for 45 CRC cases, 457 pairs of cases and controls were enrolled in this study. Additionally, because 29 blood samples from cases or controls were not successfully tested, 428 pairs of cases and controls were eventually included in the final analysis. All subjects were Han Chinese. The basic characteristics of the CRC cases and controls are shown in **Supplementary Table 1**. All participants provided written informed consent prior to participation in the study. Blood samples were collected before surgery for the CRC patients and before any therapy for the controls. This study was approved by the Ethics Committee of HMU.

All CRC patients had operable stage I-IV CRC, and their CRC diagnosis was histologically confirmed by a senior surgeon (YYL). Patients with adenomatous polyposis coli, who had a family history of CRC in first-degree relatives according to the Amsterdam criteria (22), or who received anti-cancer therapy before surgery were excluded.

##### 2.1.1.2 *IGF2* methylation in PBLs and CRC prognosis

Among the eligible 428 CRC cases with blood samples, 281 cases were diagnosed at the Cancer Hospital of HMU and 147 cases at the Second Affiliated Hospital of HMU. Because of the lack of follow-up data of the 147 cases from the Second Affiliated Hospital of HMU, these 147 cases were excluded from the prognosis analysis. Eventually, only the 281 cases from the Cancer Hospital of HMU were included in the prognostic analysis. Fasting venous blood samples for methylation testing and leukocyte count testing were collected in the morning of a day before surgery. The peripheral leukocyte cells were counted by Sysmex XT-1800 Automated Hematology System (Shanghai, China). For each patient, extensive demographic, clinicopathological and treatment information were extracted from the electronic medical record system. All surgical operations were performed by the same surgical oncologist (YLL) and all patients had negative surgical margins. The basic characteristics of the CRC patients included in this cohort are shown in **Supplementary Table 2**.

The primary outcomes were overall survival (OS) from CRC diagnosis to death and disease free survival (DFS) from CRC diagnosis to disease recurrence, metastasis, or death from CRC, whichever came first. Another outcome was CRC-specific survival (CSS), which was defined as the time from disease diagnosis to CRC-specific death. Outcomes were observed during the follow-up period through March 15, 2014 *via* an established protocol. Postoperative patients were followed up at 3-6 months intervals for the first year and then annually. We used a telephone-delivery follow-up questionnaire to collect information on the date and cause of death of the CRC patients. The recorded date and cause of death of the CRC patients were validated using the medical certification of death and the Harbin death registration system. We excluded those patients with less than 30 days of follow-up in the prognosis analysis. Of the 281 eligible CRC patients included in this analysis, 127 died, 120 remained alive and 34 were lost to follow-up. The interval between CRC diagnosis and outcomes ranged from 31 to 3335 days, with a median value of 2123 days (interquartile range: 996-2431 days).

##### 2.1.1.3 *IGF2* methylation in tumour tissues and CRC prognosis

In order to analyze the relationship between *IGF2* methylation in tumor tissues and CRC prognosis, we collected fresh tumor tissues from 310 patients at the Cancer Hospital of HMU. Because 13 tissue samples were not successfully tested, 297 cases were eventually included in this prognosis analysis. Among the 297 eligible CRC patients included in this analysis, 124 died, 141 remained alive and 32 were lost to follow-up. The interval between CRC diagnosis and outcomes ranged from 31 to 3335 days, with a median value of 2251 days (interquartile range: 1218-2424 days). The CRC patients who provided tissue samples and the patients who provided blood samples were from different cohorts. In all, 185 paired tumour tissue and PBL samples were obtained from the same patients.

##### 2.1.1.4 Sample size

The sample size was estimated using PASS version 11.0.7 (NCSS LLC., USA). To assess whether aberrant methylation of *IGF2* in peripheral blood leukocytes (PBLs) is associated with the risk of developing colorectal cancer (CRC) in our present case-control study, we estimated the sample size according to a logistic regression model. A sample size of 652 participants was needed to achieve 90% power (at the 5% level of statistical significance) in order to detect an odds ratio (OR) of 1.8 or more with a 20% prevalence in the control group. In addition, taking into consideration incomplete questionnaires and the failure rate for MS-HRM detection, we included approximately 20% more patients and finally targeted a total sample size of 800 participants (n = 400 for CRC patients and controls, respectively).

To assess whether aberrant *IGF2* methylation in PBLs is associated with CRC patient prognosis in our present cohort study, we estimated the sample size according to a Cox regression model. A sample size of 184 CRC patients was needed to achieve 80% power (at the 5% level of statistical significance) to detect a hazard ratio (HR) of 0.5 or 2.0 with an overall event rate of 50% in this cohort. In addition, taking into consideration incomplete questionnaires and the failure rate for MS-HRM detection, we included approximately 20% more patients and finally targeted a total sample size of 221 CRC patients.

### 2.2 Validation study

A case-control study nested in the EPIC-Italy cohort (GSE51032) and a twins’ case-control study (GSE89093) from the GEO were used to validate the association between PBL *IGF2* methylation and CRC risk. We also used the EPIC-Italy CRC cohort and TCGA datasets (COAD and READ) to validate the relationship between *IGF2* methylation in PBLs or tumour tissues and CRC prognosis, respectively. The basic characteristics of validation populations are shown in **Supplementary Tables 1**, **2**. Methylation levels were detected using the 450k array in all validation datasets, which were downloaded from the GEO (https://www.ncbi.nlm.nih.gov/gds/) and UCSC Xena (https://xenabrowser.net/datapages/) websites. We directly extracted the already processed β-values (representing their methylation levels) for each CpG locus from these datasets. We then calculated the average β-value of these six CpG loci as the regional methylation level for subsequent validation analysis. In each validation dataset, we divided the study population into hypermethylation and hypomethylation groups based on the median methylation level.

### 2.3 Methylation analysis

Sample processing, DNA extraction and bisulfite modification were performed as described previously (20). Briefly, DNA was extracted from buffy coats and tumour tissues using a QIAamp DNA Blood Mini Kit (Qiagen, Hilden, Germany, Cat#51106) and a DNeasy Blood & Tissue Kit (Qiagen, Cat #69506), respectively. The DNA was then bisulfite-modified using an EpiTect Plus DNA Bisulfite Kit (Qiagen, Cat#59826) according to the manufacturer’s protocols. The bisulfite-modified DNA sample was quantified using a NanoDrop 2000c bioanalyzer (Thermo-Fisher, USA), diluted to a final concentration of 10 ng/µL and divided into aliquots for storage (-20°C).

**Figure 2:**
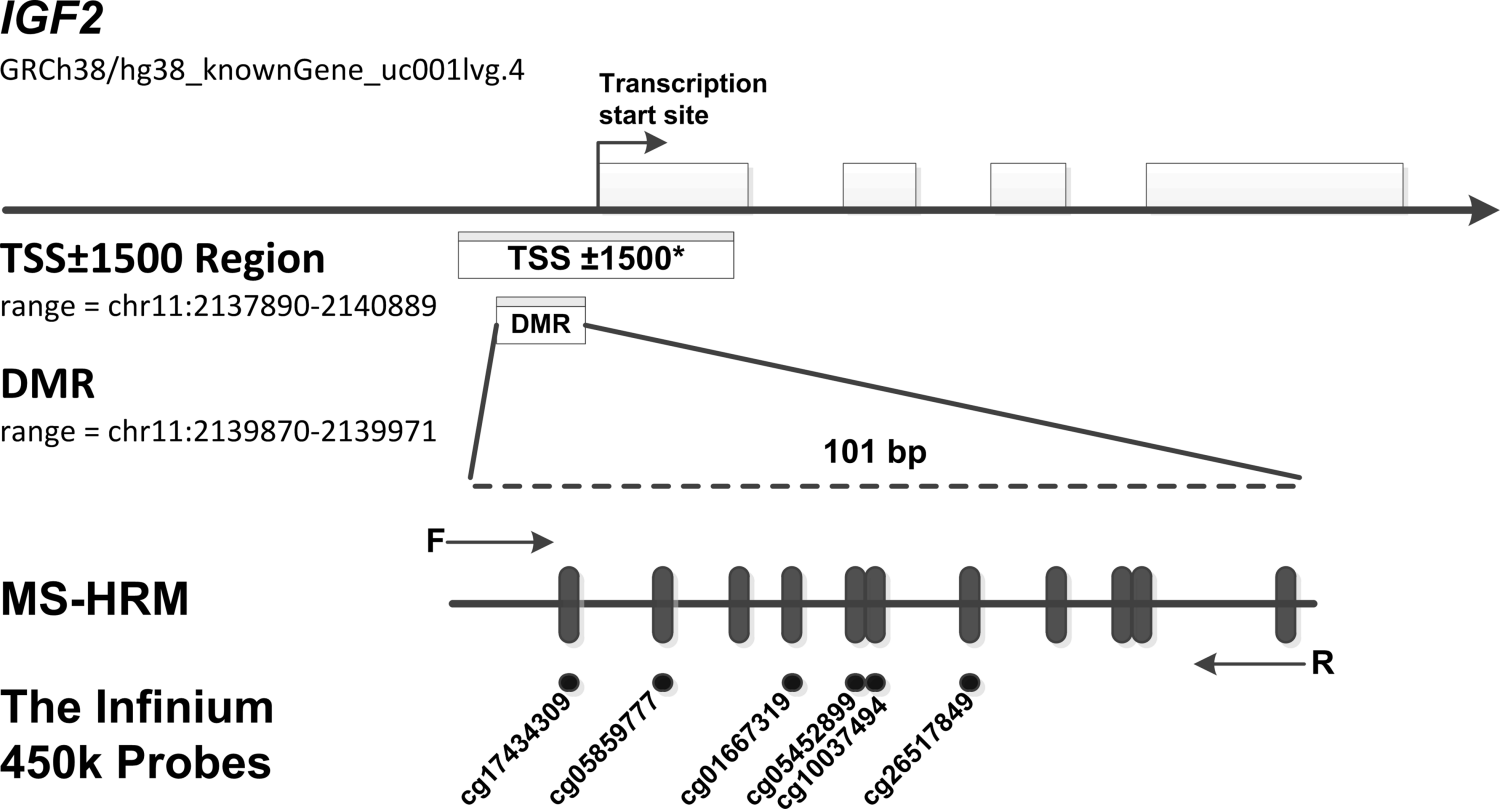
MS-HRM assay to detect methylation levels at the *IGF2* promoter region. Our assay encompassed a 101 bp region (range: chr11: 2139870-2139971, including 11 CpGs), which located in the promoter of human *IGF2* gene and near the transcription start site (TSS). Arrows indicate position and direction of MS-HRM primers. Black dashes (|) indicate individual CpGs on the *IGF2* promoter region tested in our study. Black Dots (•) indicate six methylation probes (including cg17434309, cg05859777, cg01667319, cg05452899, cg10037494, cg26517849) on the Illumina Infinium HumanMethylation450 BeadChip, which overlap CpG sites detected in our tested region. *The TSS ± 1500 region is an expanded region of -1500 bp upstream to +1500 downstream from the TSS (range: chr11: 2137890-2140889). To test the robustness of our results, we further assess the association of methylation levels of this expanded region with CRC risk. In the TSS ± 1500 region, a total of 32 CpG sites were detected by the Infinium HumanMethylation450 methylation probes. The methylation probes are as follows: cg25163476, cg24917382, cg24366657, cg23905216, cg23676551, cg22287492, cg21667878, cg20339650, cg19443075, cg19371526, cg19002337, cg18087943, cg17434309, cg17037101, cg16415340, cg15393937, cg14608156, cg14188639, cg13756879, cg12773325, cg12614029, cg11915650, cg10659464, cg10037494, cg09694722, cg08162473, cg05859777, cg05452899, cg04072545, cg03760951, cg02835822, cg01667319.

We designed a methylation-sensitive high-resolution melting (MS-HRM) assay for the *IGF2* promotor region (101bp, GRCh38/hg38; chr11:2139870-2139971, including 11 CpG sites, **Figure 2**) using Methprimer (23), and *IGF2* methylation status was tested with the researchers being blinded to patient outcome. A set of methylation standards (100, 25, 10, 5, 2, 1, and 0% methylated DNA) were prepared by mixing commercially available methylated and unmethylated DNA (Zymo Research, Irvine, USA, Cat#D5014); these standards were used to semi-quantitatively measure the methylation level of the *IGF2* target region in the samples. The MS-HRM analysis was performed as previously described. Briefly, each PCR mixture had a total volume of 10 µL and contained 2× LightCycler 480 High Resolution Melting Master Mix (Roche Applied Science, Mannheim, Germany, Cat#4909631001), 3 mmol/L MgCl2, 0.4 µmol/L of each primer (forward primer: GGGATTTGGTTGAGGTTTTAAG; reverse primer: TACGACTAAAAAAACCCCTAAACTC) and 1 µL (approximately 10 ng) of bisulfite-modified template DNA. PCR conditions were as follows: initial PCR activation (95°C for 15 minutes); 55 3-step cycles (95°C for 10 seconds, 56°C for 30 seconds, and 72°C for 20 seconds); and final extension (72°C for 10 minutes). A blank control (non-template control) sample was included in each batch, and all reactions were performed in duplicate (as technical replicates). A third trial was conducted for the samples that presented inconsistent results between the two trials. PCR amplification and MS-HRM analyses were performed using the LightCycler 480 platform (Roche). After normalization of the melting curves using the software module of Gene Scanning (Roche), two investigators (YP.L. and HR.S.), who were blinded to the sample groups, independently assessed the MS-HRM data. Discrepancies were resolved by discussion and consensus with another investigator (YBN.W.).

### 2.4 Covariates, missing data analysis and imputation

All participants were interviewed face-to-face to complete a structured standard questionnaire, which was partially adopted from Shu et al. (24) The questionnaire queried information on demographic characteristics, lifestyle factors (including family history, smoking, alcohol consumption, occupational physical activity), and diet. Dietary consumption over the past year was assessed using a validated food frequency questionnaire (FFQ) (24). The FFQ included nine major food groups, which represented most of the common foods in Northeast China. The food items included barbecued foods, coarse grains, fish stewed with brown sauce, fresh fruits, fried foods, green vegetables, leftovers and pork.

Questionnaire-derived covariates included: age (< 60, ≥ 60), gender (male, female), BMI (< 24, ≥ 24), family history of cancer other than CRC in first-degree relatives (no, yes), occupational physical activity (blue-collar, white-collar), smoking status (no, yes), and consumption of barbecued foods (< 1, ≥ 1 times/week), coarse grains (< 50, ≥ 50 g/week), fish stewed with brown sauce (< 1, ≥ 1 times/week), fruits (< 2, ≥ 2 times/week), fried foods (< 1, ≥ 1 times/month), green vegetables (< 100, ≥ 100 g/day), leftovers (< 1, ≥ 1 times/week) and pork (< 250, ≥ 250 g/week). All questionnaire-derived variables were analysed *via* missing value analysis and were imputed *via* multiple imputations as described in our previous study (20).

### 2.5 Statistical analysis

Means and standard deviations were reported for continuous variables, while counts and frequencies were reported for categorical variables. Covariate differences between groups were compared using the standardised differences method with a significant imbalance level of standardised difference ≥ 25%. In the CRC risk analysis, we first categorised individuals into two groups according to the optimal cut-off point for the *IGF2* methylation level (≤ 1% hypomethylation group; > 1% hypermethylation group), which was determined using the receiver operating characteristic (ROC) curve and the Youden index method with case-control status as the dependent variable (0 for controls; 1 for cases). We then conducted univariate and multivariate logistic regression analyses and reported odd ratios (ORs) and 95% confidence intervals (CIs) to assess the association between *IGF2* methylation status and CRC risk. In the CRC prognosis analysis, the cut-off point for the *IGF2* methylation level was also 1% using the same method with overall survival time as the dependent variable (0 for less than median survival time; 1 for longer than or equal to median survival time). According to this cut-off point, CRC patients were categorised into *IGF2* hypomethylation and *IGF2* hypermethylation groups (206 and 75 cases, respectively). Kaplan-Meier curves and log-rank tests were used to compare OS, DFS or CSS between groups. The associations between *IGF2* methylation and OS, DFS or CSS were estimated using univariate and multivariate Cox regression models and reported as hazard ratios (HRs) and 95% CIs. Two-sided statistical significance was defined as *P*<0.05. ROC analyses were performed with MedCalc version 15.4 (Ostend, Belgium) and all other statistical analyses were performed with SPSS Statistics version 23.0 (IBM, Inc., USA).

To minimise group differences on covariates, we performed a PS-based analysis. In the CRC risk analysis, the PS was calculated with case-control status as the dependent variable using a multivariate logistic regression model that included demographic and lifestyle factors. Before PS adjustment, the basic characteristics of the subjects differed significantly between the case and control groups; after PS adjustment, these differences were significantly lower and well balanced (**Supplementary Table 3**). In the survival analysis, the PS was calculated with *IGF2* methylation as the dependent variable using a multivariate logistic regression model that included demographic and lifestyle factors and clinicpathologic characteristics. Similarlly, PS adjustment also significantly reduced the difference of the charcteristics at baseline between the *IGF2* hypermethylation and hypomethylation groups among CRC patients (**Supplementary Table 4**). To incorporate all the patients in the analyses, we primarily employed the PS-adjustment method (14). Additionally, several PS-based methods, including stratification by quintile of PS, weighting with inverse probability of treatment weights, and individual PS matching, were also performed as sensitivity analyses.

For PS-stratification analyses, all patients were stratified by PS quintile. HRs for PS-stratified analyses were obtained by pooling effect estimates from each PS quintile. For PS-weighted analyses, each patient was weighted by the inverse probability of being in the higher versus lower *IGF2* methylation groups; this method is known as weighting with inverse probability of treatment weights (IPTW). The weight for each patient was calculated according to the method described by Robins and Hernan (25): Pt/PS and (1-Pt)/(1-PS) for patients in the *IGF2* hypermethylation and hypomethylation groups, respectively; where Pt is the proportion of the patients with *IGF2* hypermethylation in all participants. In addition to using PS as an adjustment or weighting factor in the analysis model, PS was also used for individual matching. PS matching was performed using a nearest-neighbour algorithm with a calliper of 0.2 (which means that the maximum allowable PS difference between groups was no larger than 20% of the standard deviation of PS).

### 2.6 Sensitivity analysis

To assess the robustness of our results, we performed extensively predefined sensitivity analyses. First, to evaluate the potential impact of the PS-adjusted confounders on our results in the initial study, we performed confounding ratio of risk ratio (RR) analysis, which was defined as the ratio of the PS-adjusted effect estimates and the unadjusted effect estimates (26). To investigate whether potential residual confounders could impact the results, we calculated the E-value for PS-adjusted effect estimates and the limit of the CI closest to the null (27). Finally, we performed subgroup analyses according to the tumour location (colon or rectum), UICC stage, gender (female vs. male), age (≥60 vs. <60 years), and body mass index (BMI, ≥24 vs. <24).

Additionally, we performed several *post hoc* sensitivity analyses. To test whether tumour load impacts *IGF2* methylation level in PBLs, we assessed the associations between *IGF2* methylation and UICC stage and serum carcinoembryonic antigen (CEA) level. We also tested whether PBL *IGF2* methylation levels could have been impacted by leukocyte count and the percentage of certain subpopulations. In addition, to comprehensively determine the association between *IGF2* methylation and CRC risk, we combined the results from the initial case-control and the validation studies using a meta-analysis method of random effect model. Finally, we explored whether PBL *IGF2* methylation levels differ between the CRC patients included in the survival analysis and those excluded.

## 3 Results

### 3.1 *IGF2* methylation in PBLs and CRC risk

**Figure 3:**
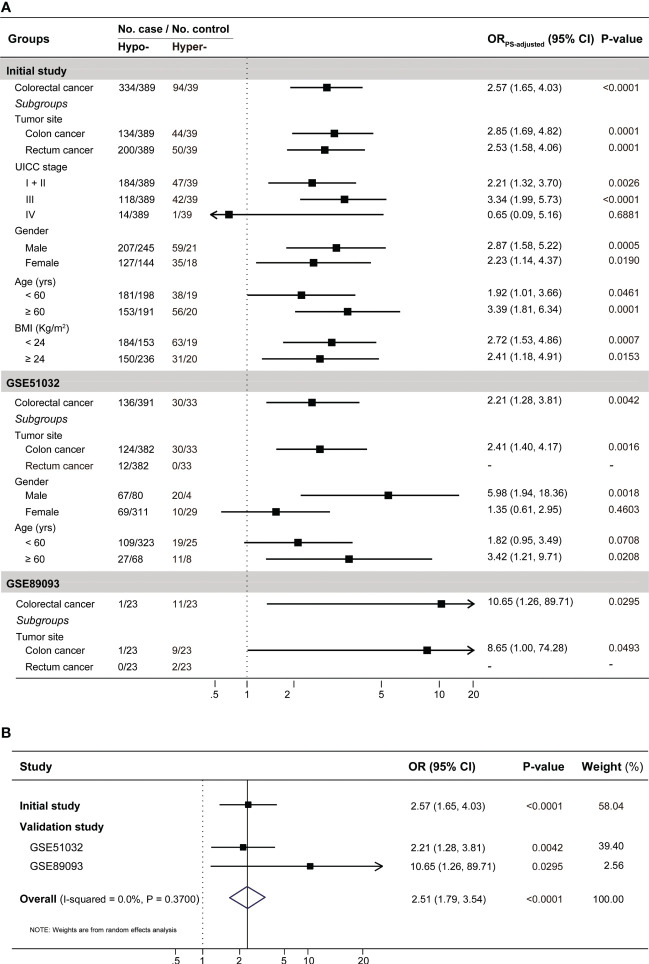
The results for the association between PBL *IGF2* methylation and the risk of developing CRC **(A)** in the initial and validation studies, and **(B)** in the combined populations. A total of 14 covariates were included in the propensity score model in the initial case-control study (see **Supplementary Table S3**). In the validation studies, gender and age were included in the the propensity score model for GSE51032 and age for GSE89093, respectively. To be conservative, we pooled the results from these three datasets with a random effect model in the meta-analysis. BMI, body mass index; CI, confidence interval; Hyper-, *IGF2* hypermethylation; Hypo-, *IGF2* hypomethylation; OR, odds ratio.

As shown in **Figure 3A**, subjects with *IGF2* hypermethylation (15.54%), compared to subjects with *IGF2* hypomethylation (84.46%), had a significantly increased CRC risk (PS-adjusted OR, 2.57, 95% CI: 1.64-4.03; *P*<0.001). This association remained statistically significant even after subgroup analyses. Notably, stratified analyses showed a positive association only in UICC stage I-III but not in stage IV cancers.

**Figure 4:**
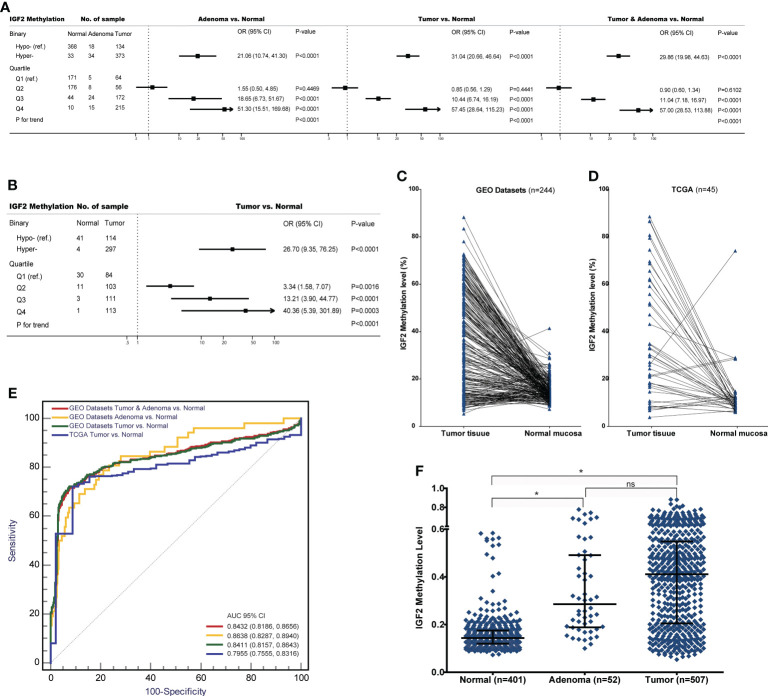
The associations between *IGF2* methylation in tissue samples and CRC risk in the datasets from GEO and TCGA. **(A)** Adenoma or tumour tissues versus normal colorectal mucosa in the GEO datasets. **(B)** Tumour tissues versus normal colorectal mucosa in the TCGA datasets. **(C)** Tumour tissues versus matched adjacent normal tissues in the datasets from **(C)** GEO and **(D)** TCGA. **(E)** The discrimination performance of *IGF2* methylation in tissue samples to identify CRC tumour or adenoma tissue and normal colorectal mucosa in the datasets from GEO and TCGA. **(F)** The *IGF2* methylation levels in normal colorectal mucosa, adenoma and tumour tissue samples in the GEO datasets. *indicates *P*<0.0001, and ns indicates not statistical significance. AUC, the area under receiver operating characteristic curves; CI, confidence interval; OR, odds ratio; Hyper-, *IGF2* hypermethylation; Hypo-, *IGF2* hypomethylation; ns, not statistical significance.

In the EPIC-Italy dataset, *IGF2* hypermethylation was also significantly associated with an increased CRC risk (OR, 2.21, 95% CI: 1.28-3.81; *P*=0.004). Subgroup analyses showed the association was statistically significant in distal colon cancer, men, and older subjects (**Figure 3A**). Using the GSE89093 dataset, *IGF2* hypermethylation was strongly associated with an increased risk of CRC or colon cancer alone. After pooling the results from these three datasets, the observed association was still significant (**Figure 3B**). In addition, the results from the GEO and TCGA tumor tissue datasets also supported the association between *IGF2* hypermethylation and the risk of developing CRC (**Figure 4**).

### 3.2 *IGF2* methylation in PBLs and CRC prognosis

**Figure 5:**
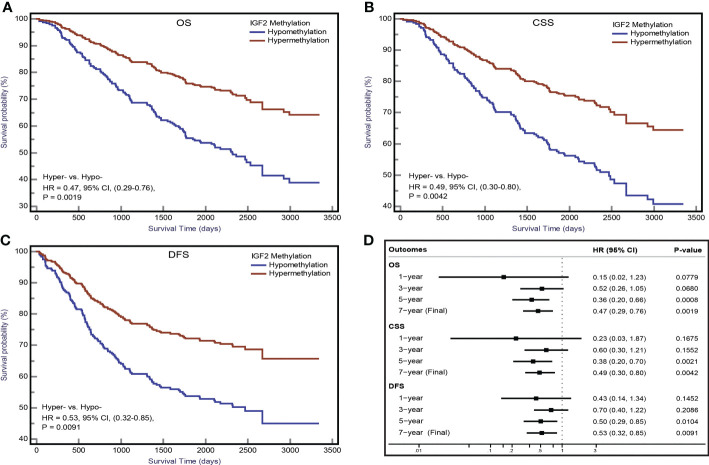
*IGF2* methylation in PBLs and CRC prognosis in the initial cohort. Kaplan-Meier plots for **(A)** overall survival, **(B)** CRC specific survival, and **(C)** disease-free survival according to *IGF2* methylation status in PBLs in CRC patients. **(D)** Associations of *IGF2* methylation in PBLs with CRC prognosis during different follow-up periods. CI, confidence interval; CRC, colorectal cancer; CSS, cancer specific survival; DFS, disease free survival; HR, hazard ratio; Hyper-, *IGF2* hypermethylation; Hypo- *IGF2* hypomethylation; OS, overall survival; PBL, peripheral blood leukocyte.

The Kaplan-Meier survival curves for the PBL *IGF2* hypermethylation and hypomethylation groups are shown in **Figure 5**. The median OS was 73 months (95% CI: 66-80 months) in the *IGF2* hypermethylation group versus 56 months (95% CI: 52-61 months) in the hypomethylation group. The OS rate was 75% (95% CI: 60-80%) in the *IGF2* hypermethylation group versus 62% (95% CI: 50-57%) in the hypomethylation group (HR_PS-adjusted_, 0.47, 95% CI: 0.29-0.76; *P*=0.002). The CSS rate was 56% (95% CI: 45-68%) in the *IGF2* hypermethylation group versus 38% (95% CI: 31-45%) in the hypomethylation group (HR_PS-adjusted_, 0.49, 95% CI: 0.30-0.80; *P*=0.004). The median DFS was 66 months (95% CI: 57-74 months) in the *IGF2* hypermethylation group versus 52 months (95% CI: 48-57 months) in the hypomethylation group (HR_PS-adjusted_, 0.53, 95% CI: 0.32-0.85; *P*=0.009). Based on subgroup analyses, we found that the statistically significant association persisted in rectal cancers, UICC stage I-III cancers, males, older, or normal body weight patients. In contrast, the effect estimates did not reach statistical significance in colon cancers, stage IV cancers, females, younger, and overweight or obese patients (**Table 1**).

**Table 1:** Subgroup analysis for the association of PBL *IGF2* methylation with CRC prognosis.

Subgroups		Univariate HR, 95% CI	P-value	PS-adjusted HR, 95% CI	P-value	P-value for heterogeneity*
Tumour Site						
OS	Colon	0.65 (0.33-1.27)	0.2049	0.59 (0.29-1.17)	0.1309	0.4117
	Rectum	0.49 (0.28-0.86)	0.0132	**0.39 (0.20-0.77)**	**0.0065**	
CSS	Colon	0.60 (0.29-1.21)	0.1545	0.55 (0.27-1.13)	0.1062	0.6706
	Rectum	0.53 (0.30-0.94)	0.0293	**0.45 (0.22-0.88)**	**0.0204**	
DFS	Colon	0.51 (0.25-1.05)	0.0661	0.48 (0.23-1.00)	0.0514	0.7539
	Rectum	0.68 (0.39-1.18)	0.1714	0.56 (0.29-1.09)	0.0895	
UICC Stage						
OS	I + II + III	0.58 (0.37-0.90)	0.0158	**0.45 (0.28-0.74)**	**0.0017**	
	I + II	0.57 (0.27-1.21)	0.1427	**0.35 (0.15-0.82)**	**0.0155**	0.5840
	III	0.54 (0.31-0.95)	0.0327	**0.54 (0.30-0.98)**	**0.0440**	
	IV	1.04 (0.13-8.38)	0.9726	0.94 (0.11-8.08)	0.9542	
CSS	I + II + III	0.59 (0.37-0.93)	0.0243	**0.47 (0.29-0.78)**	**0.0037**	
	I + II	0.59 (0.27-1.30)	0.1880	**0.39 (0.16-0.97)**	**0.0427**	0.7135
	III	0.55 (0.31-0.97)	0.0375	**0.54 (0.30-0.98)**	**0.0441**	
	IV	1.04 (0.13-8.38)	0.9726	0.94 (0.11-8.08)	0.9542	
DFS	I + II + III	0.64 (0.40-1.00)	0.0508	**0.51 (0.31-0.84)**	**0.0084**	
	I + II	0.63 (0.29-1.37)	0.2435	**0.41 (0.17-0.99)**	**0.0471**	0.7337
	III	0.61 (0.35-1.06)	0.0812	0.60 (0.33-1.10)	0.0974	
	IV	0.67 (0.08-5.34)	0.7048	0.75 (0.09-6.55)	0.7982	
Gender						
OS	Female	0.72 (0.39-1.32)	0.2910	0.50 (0.24-1.04)	0.0626	0.6172
	Male	0.40 (0.21-0.76)	0.0054	**0.39 (0.20-0.77)**	**0.0066**	
CSS	Female	0.74 (0.39-1.40)	0.3552	0.55 (0.26-1.16)	0.1163	0.5527
	Male	0.41 (0.22-0.79)	0.0071	**0.40 (0.20-0.79)**	**0.0086**	
DFS	Female	0.75 (0.40-1.42)	0.3789	0.55 (0.26-1.16)	0.1184	0.7691
	Male	0.49 (0.26-0.90)	0.0226	**0.48 (0.25-0.92)**	**0.0277**	
Age, (years)						
OS	< 60	0.54 (0.27-1.06)	0.0719	0.53 (0.26-1.11)	0.0948	0.6705
	≥ 60	0.54 (0.30-0.95)	0.0339	**0.43 (0.23-0.81)**	**0.0082**	
CSS	< 60	0.55 (0.28-1.08)	0.0804	0.56 (0.27-1.17)	0.1211	0.6533
	≥ 60	0.56 (0.31-1.01)	0.0555	**0.45 (0.23-0.85)**	**0.0144**	
DFS	< 60	0.60 (0.30-1.18)	0.1379	0.68 (0.30-1.30)	0.2123	0.5624
	≥ 60	0.59 (0.33-1.05)	0.0747	**0.47 (0.25-0.89)**	**0.0197**	
BMI, (Kg/m2)						
OS	< 24	0.52 (0.30-0.88)	0.0144	**0.47 (0.27-0.85)**	**0.0119**	0.9947
	≥ 24	0.56 (0.25-1.23)	0.1473	0.48 (0.21-1.07)	0.0726	
CSS	< 24	0.56 (0.33-0.97)	0.0370	**0.53 (0.29-0.95)**	**0.0333**	0.7016
	≥ 24	0.49 (0.21-1.14)	0.0984	0.43 (0.18-1.02)	0.0557	
DFS	< 24	0.61 (0.36-1.04)	0.0689	**0.54 (0.30-0.97)**	**0.0378**	0.8742
	≥ 24	0.51 (0.22-1.20)	0.1234	0.49 (0.21-1.18)	0.1127	

*Test for heterogeneity between ORs was conducted by using random effect models with Comprehensive Meta Analysis (version 2.2.046). BMI, body mass index; CI, confidence interval; CRC, colorectal cancer; CSS, cancer specific survival; DFS, disease free survival; HR, hazard ratio; OS, overall survival; PBL, peripheral blood leukocyte; PS, propensity score.

The bold values represent P-value < 0.05.

**Figure 6:**
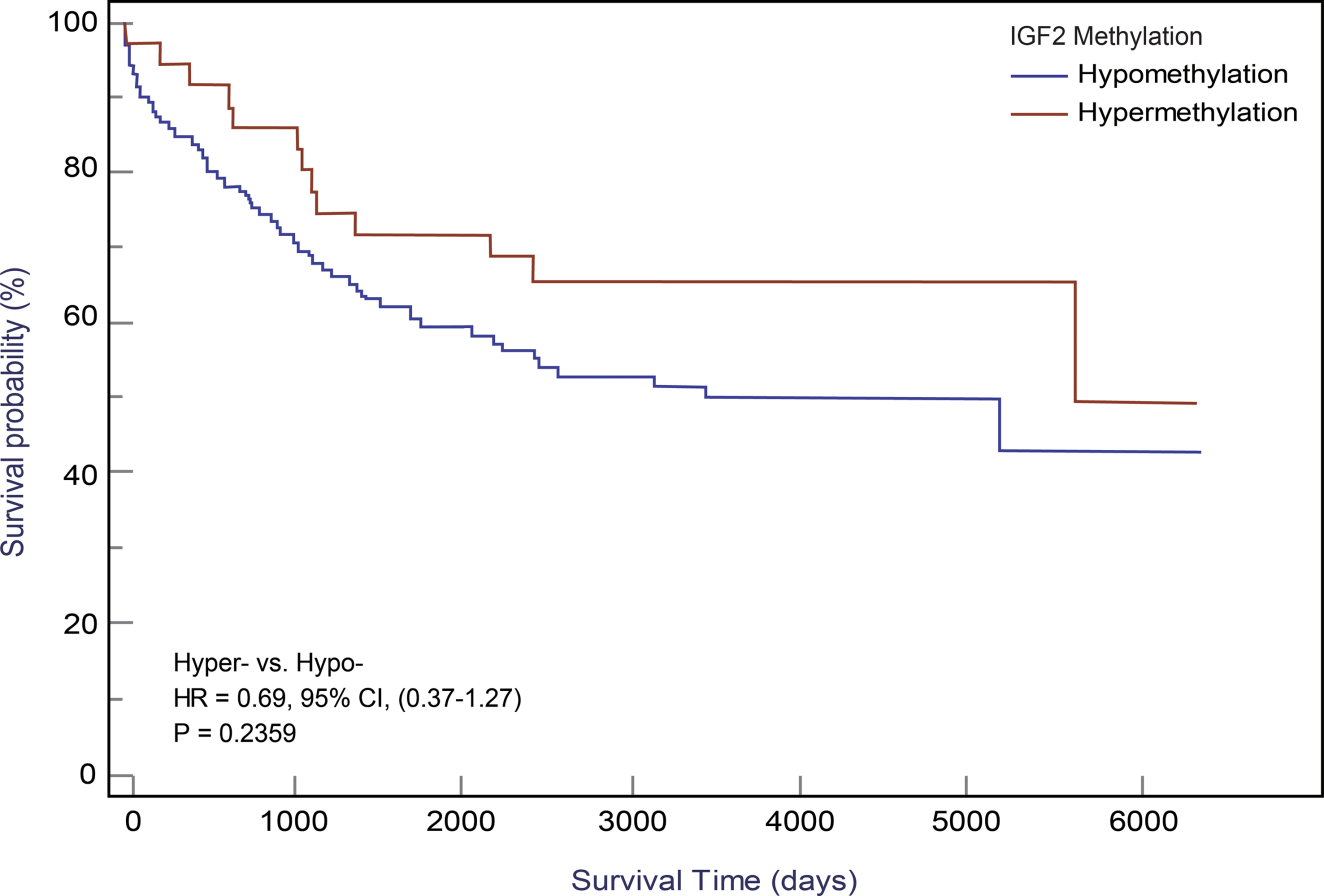
Kaplan-Meier curves for *IGF2* methylation in PBLs and CRC overall survival in the EPIC-Italy cohort. CI, confidence interval; HR, hazard ratio; Hyper-, *IGF2* hypermethylation; Hypo- *IGF2* hypomethylation.

Using the follow-up results of the 166 CRC patients from the EPIC-Italy cohort, we observed a clear trend for a longer OS related with *IGF2* hypermethylation, although the association was not statistically significant. The univariate HR was 0.65 (95% CI: 0.35-1.19; *P*=0.164);, and after PS adjustment, the HR_PS-adjusted_ was 0.69 (95% CI: 0.37-1.27; *P*=0.236; K-M survival curves are shown in **Figure 6**).

### 3.3 *IGF2* methylation in tissues and CRC prognosis

**Figure 7:**
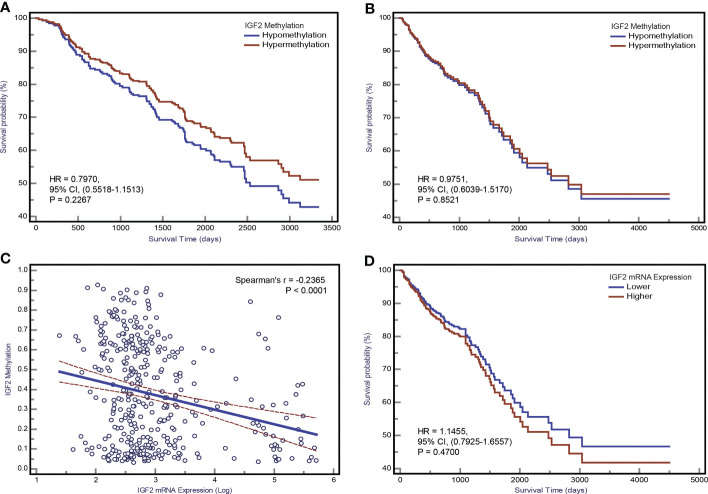
The association between *IGF2* methylation in tumour tissues and CRC patient prognosis. Kaplan-Meier curves for overall survival according to *IGF2* methylation status in tumour tissues of CRC patients in **(A)** the initial cohort and **(B)** TCGA datasets. **(C)** The correlation between *IGF2* mRNA expression level and methylation level of the differentially methylated region of *IGF2* tested in our study using the TCGA dataset. **(D)** Kaplan-Meier curves for overall survival according to *IGF2* mRNA expression levels in tumour tissues of CRC patients in the TCGA cohort. CI, confidence interval; CRC, colorectal cancer; HR, hazard ratio.

In our initial study, the association between *IGF2* methylation in tumour tissues and CRC patient survival did not reach statistical significance (**Figure 7A**), and this result was confirmed by the TCGA dataset (**Figure 7B**). Using *IGF2* mRNA expression data in tumour tissues, we found an obvious negative correlation with *IGF2* methylation (r=-0.24, *P*<0.001; **Figure 7C**). So we further explored whether *IGF2* mRNA expression levels impact the disease prognosis but found no statistically significant association (**Figure 7D**).

### 3.4 Sensitivity analysis

**Figure 8:**
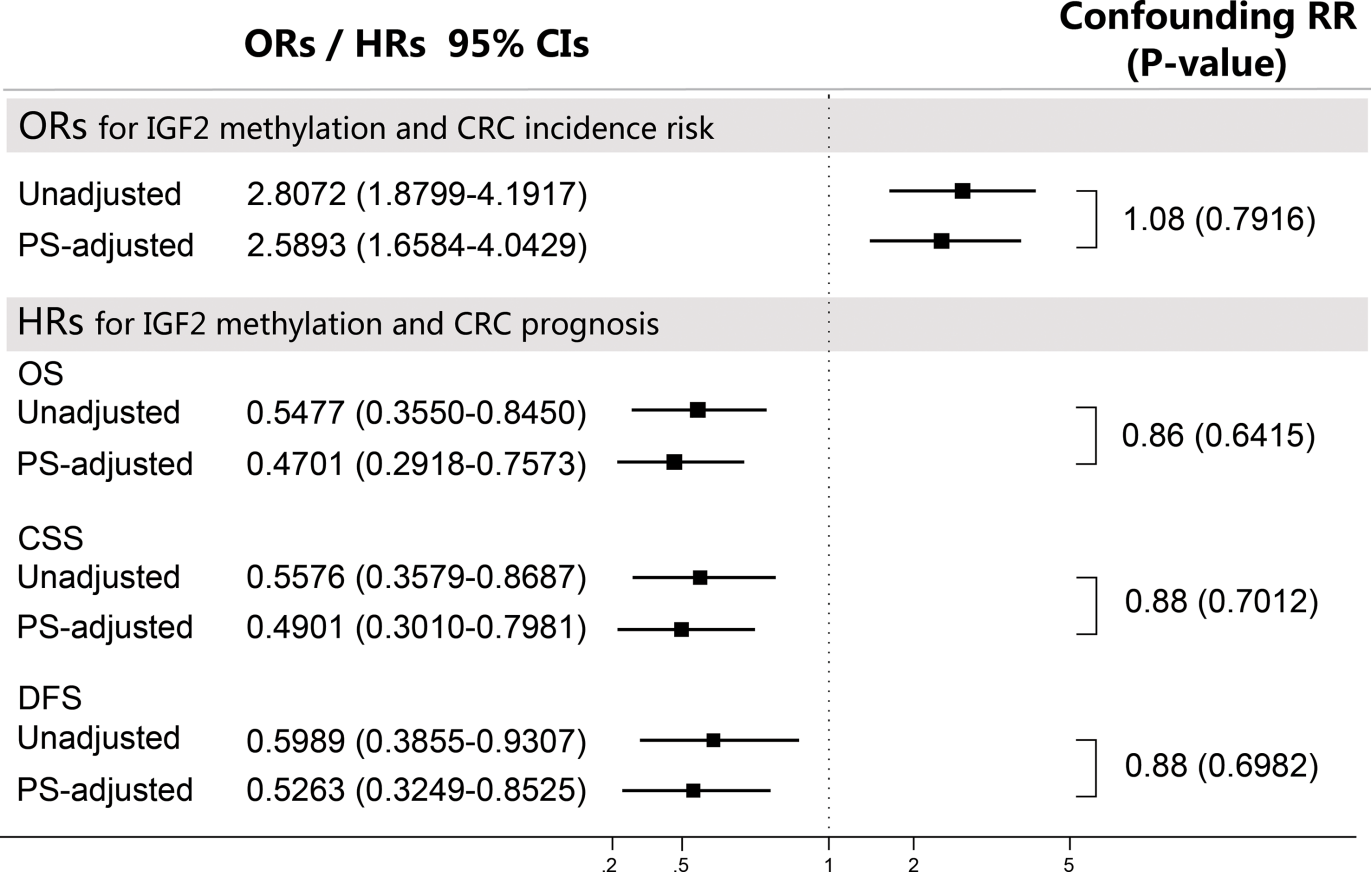
Sensitivity analyses using the confounding RR method. The confounding RR was defined as the ratio of the PS-adjusted effect estimates and the unadjusted effect estimates. CI, confidence interval; CRC, colorectal cancer; CSS, cancer specific survival; DFS, disease free survival; HR, hazard ratio; OR, odds ratio; OS, overall survival; PS, propensity score.

We repeated analyses using other PS-based methods and the results were consistent with the PS-adjustment results (**Supplementary Tables 5**, **6**). Based on the confounding RR analysis (**Figure 8**), we did not find any substantial differences between the PS-adjusted effect estimates and the corresponding unadjusted effect estimates. Furthermore, the E-value analysis showed that our findings in both the initial and validation studies appear to be very robust (**Supplementary Table 7**).

**Figure 9:**
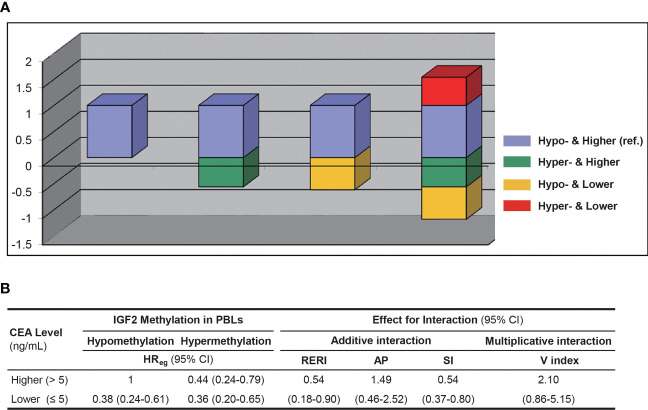
The interaction between PBL *IGF2* methylation and serum CEA levels on CRC patient prognosis using the additive and multiplicative models. **(A)** The interaction analysed with the additive model. **(B)** The effect estimates for interaction in respected to both the additive and multiplicative models. AP, attributable proportion due to interaction; CI, confidence interval; HR, hazard ratio; Hyper-, *IGF2* hypermethylation; Hypo-, *IGF2* hypomethylation; PBL, peripheral blood leukocyte; RERI, relative excess risk due to interaction; SI, synergy index; V index, the multiplicative interaction index.

We found no evidence for an obvious impact of UICC stages on *IGF2* methylation in PBLs (**Supplementary Table 8**). Of note, *IGF2* hypermethylation was significantly more frequent in the CEA-low group than in the CEA-high group (34.15% vs. 20.89%, *P*=0.013). We found no significant relationship between the PBL *IGF2* methylation levels and the leukocyte count or the percentages of certain leukocyte subpopulations. We further performed a sensitivity analysis by including the leukocyte count data in the PS model and found similar results (**Supplementary Table 9**). Another sensitivity analysis using the additive model showed that after adjustment for CEA and the interaction between *IGF2* methylation and CEA, the effect of *IGF2* hypermethylation itself on CRC prognosis remained statistically significant (**Figure 9**). Finally, we analysed samples from 147 additional CRC patients that were excluded from the survival analysis and found no differences in their PBL *IGF2* methylation levels compared to those of the 281 CRC patients included in the survival analysis (**Supplementary Table 8**).

## 4 Discussion

In this study, we first assessed the impact of the PBL IGF2 methylation status on the risk and prognosis of CRC. We found that individuals with IGF2 hypermethylation in their PBLs were at significantly higher risk of developing CRC than those with IGF2 hypomethylation. However, our findings showed better survival rates in CRC patients with PBL IGF2 hypermethylation compared to those in CRC patients with IGF2 hypomethylation.

In our initial case-control study, it is not possible to determine the aetiologically relevant time window of IGF2 methylation relative to CRC development. Fortunately, the prospective nature of the EPIC-Italy cohort is invaluable for confirming the temporal sequence of DNA methylation and CRC onset and therefore helps to distinguish causal from consequential epigenetic changes (28, 29). In this nested case-control study, blood samples from CRC patients were collected 0.02-14.40 years (average 6.16 years) before diagnosis. Using this dataset, we found a positive but non-significant association between PBL IGF2 hypermethylation and an increased CRC risk. To account for reverse causality, we repeated the analyses excluding subjects who developed CRC within two years after their blood sampling and found that this positive association remained significant (PS-adjusted OR, 2.08, 95% CI: 1.18-3.69; *P*=0.012). Furthermore, our main finding was verified using the GSE89093 dataset. Finally, we pooled the results from these three datasets and found a 2.19-fold higher risk of developing CRC in the *IGF2* hypermethylation group compared to the hypomethylation group.

The exact mechanism that links alterations in the IGF2 methylation status in PBLs and the susceptibility for CRC remains unclear. The PBL-derived DNA methylation profiles represent the overall methylation status of an individual. Alterations in PBL-derived DNA may be constitutively present before cancer occurs or represent an early response of the haematology system to the presence of tumour cells after cancer develops. Imprinting of *IGF2* is primarily maintained by DNA methylation (30, 31). It has been reported that imprinting and expression are controlled by CpG-rich regions upstream of the *IGF2* promotors. Normally, *IGF2* is expressed from the paternal allele only, while the maternal allele is methylated and imprinted. Several previous studies have reported that the loss of imprinting (LOI) of *IGF2* in peripheral blood (leukocytes or lymphocytes) may be a potential biomarker for the risk of developing CRC (12, 32), and *IGF2* methylation alterations were suggested as a surrogate marker for LOI of *IGF2* (32, 33). If *IGF2* aberrant methylation is merely a surrogate marker for LOI of *IGF2*, then *IGF2* hypermethylation would be expected to be beneficial for maintaining the imprinting status of *IGF2* and thus should decrease the risk of developing CRC. On the contrary, our results showed that *IGF2* hypermethylation increases the risk of developing CRC, indicating that *IGF2* participates in CRC tumorigenesis through two different modes of epigenetic alteration, aberrant hypermethylation and LOI, which is supported by previous studies (34, 35). Thus, our findings demonstrate that aberrant *IGF2* hypermethylation can be assayed with a blood-based test and that the PBL *IGF2* methylation status is likely to be a valuable predictive biomarker for CRC risk, independent of LOI of *IGF2*.

Further analysis of the GEO and TCGA datasets provided additional evidence supporting the association between *IGF2* hypermethylation and the risk of developing CRC. For example, using these datasets we found significant associations between *IGF2* hypermethylation in tissues and an increased risk of CRC or adenomas (**Figures 4A–D**). Interestingly, colorectal adenomas, a precancerous condition, showed similar levels of *IGF2* methylation compared to tumour tissues (**Figures 4E, F**). Most colorectal cancers initially develop as benign precursor lesions (adenomas) that can take as long as 10 to 15 years to develop into carcinomas, which underpins early detection and removal of adenomas as an important strategy for preventing CRC (6, 36). The findings from the GEO and TCGA datasets indicate that *IGF2* methylation in tissues can discern colorectal adenomas or CRCs from normal intestinal mucosa, which also suggests that *IGF2* methylation may prove valuable during CRC screening for early cancer detection. We were unable to evaluate the association between *IGF2* methylation in PBLs and the risk of developing adenomas. This issue should be explored in future studies.

In 2008, Ito and colleagues used peripheral blood samples to assess six CpG sites located in the *IGF2* gene and reported that *IGF2* methylation is not statistically associated with CRC risk (34). However, the region tested in Ito’s study is different from the region examined in our study. As shown in **Figure 2**, the differentially methylated region assessed in our study is located near the transcription start site (TSS). To further validate our findings, we used the EPIC-Italy dataset to evaluate the methylation status of a relatively large region of -1500 bp upstream to +1500 bp downstream from the TSS. This sensitivity analysis again showed a statistically significant association between *IGF2* hypermethylation and CRC risk (OR, 1.75, 95% CI: 1.15-2.66; *P*=0.009).

For the first time, we assessed the prognostic value of PBL *IGF2* methylation status in CRC patients and found that patients with *IGF2* hypermethylation in PBLs had significantly improved survival compared to patients with *IGF2* hypomethylation. These findings were especially obvious for stage I-III CRC patients. However, this association did not reach statistical significance in the external EPIC-Italy CRC cohort. Given these inconsistent results, further cohorts with large sample size are needed to validate this novel finding.

For sensitivity analysis, we also evaluated the association between PBL *IGF2* methylation and CRC prognosis during different follow-up periods. We observed a significantly strong association for 5-year relative survival, while the associations for both 1-year and 3-year relative survival were not statistically significant. Based on these results, it is hypothesised that the beneficial effects of *IGF2* hypermethylation on CRC survival might begin the fifth year following CRC diagnosis and might persist for several years. In fact, the potentially beneficial effects may occur as early as the first year after CRC diagnosis, even though this effect did not reach statistical significance. The limited sample size of the cohort study may hinder the interpretation of these results; therefore, larger cohort studies are required to further evaluate this issue.

We also evaluated the impact of *IGF2* methylation in tumour tissues on CRC patient survival and found no statistically significant association in both our initial cohort and the TCGA datasets. This is consistent with a recent research (7). However, two previous studies from Japan have assessed the *IGF2* methylation status in tumour tissues of CRC patients and the results were inconsistent (13, 37). The region tested in those two studies is different from that examined in our study. The lack of consistency between the results from the PBL samples and tumour tissues may reflect the fact that *IGF2* methylation is merely a predictive marker rather than a prognostic marker. In addition, the detection of tissue-based markers depends on material from a biopsy or tumour tissue from resection. Because of intratumour heterogeneity, the detection of a biomarker from a single biopsy or one section of a tumour tissue sample might not necessarily represent the *IGF2* methylation status of a given patient. Repeated biopsies and tests of multiple samples, however, are not feasible in routine clinical practice. Fortunately, blood-derived biomarkers have the potential to overcome these problems. In this respect, repeat blood sampling and detection of PBL *IGF2* methylation is more acceptable and feasible than repeat biopsies in the clinic. Given that CRC has a wide range of long-term outcomes, PBL *IGF2* methylation, as a DNA-based non-invasive blood test, could prove beneficial during follow-up and help identify patients at high risk of disease recurrence and progression.

An important aspect and potential concern of using PBL DNA methylation as a biomarker is whether leukocyte subpopulations affect the methylation signature of an individual. To address this concern, we collected patient clinical records including leukocyte counts and included these data as covariates in the PS model. Theoretically, the PS adjustment would control for the potential impact of different leukocyte counts and different subpopulations on our results. We compared the results before and after including the leukocyte count data in the PS model and found similar results, suggesting that the effect of leukocyte counts and subpopulations on our results is negligible. Additional evidence supporting these findings can be found in several recently published studies which also showed that differences in leukocyte subpopulations were unlikely to interfere with the results of PBL-derived DNA methylation (38, 39).

Interestingly, our data indicates that PBL *IGF2* hypermethylation correlates with serum CEA levels before surgery. Therefore, we further assessed whether the association between PBL *IGF2* methylation and CRC prognosis is impacted by CEA levels. Using the additive model, we found a positive interaction between PBL *IGF2* methylation and CEA on CRC prognosis. Given the limited sample size of the subgroups, this aspect should be further validated in future studies. Of note, after adjustment for CEA and the interaction between *IGF2* methylation and CEA, the effect of *IGF2* hypermethylation itself on CRC prognosis remained statistically significant (HR, 0.44, 95% CI: 0.24-0.79; *P*=0.006), suggesting a robust and independent role for PBL *IGF2* hypermethylation in predicting the prognosis of CRC.

This study has several strengths. The findings from our initial studies were validated with several external datasets. In our initial CRC patient cohort, the covariates were collected prospectively and were blinded to patient outcome. We used PS techniques to control for multiple potential confounding factors. Furthermore, we performed extensive sensitivity analyses to assess the robustness of our findings. The confounding RR and the E-value sensitivity analyses demonstrated that our results are unlikely to be substantially impacted by both the adjusted confounders included in the PS models or a potential residual confounder.

In our present cohort, adjuvant chemotherapy was not offered routinely to high risk individuals. Thus, we did not analyse the clinical significance of *IGF2* methylation as a predictive biomarker for sensitivity to adjuvant chemotherapy. Further studies are needed to explore and clarify this issue. Another potential limitation is the limited sample size used in the stratified analyses. Therefore, the results from the stratified analyses should be interpreted with caution.

## 5 Conclusions

In summary, *IGF2* methylation in PBLs is significantly associated with the risk and prognosis of CRC, suggesting an important role for *IGF2* methylation as a blood-based predictive biomarker to identify of individuals at high risk of developing CRC; meanwhile, PBL *IGF2* methylation might also serve as a predictive biomarker for CRC prognosis.

## Data availability statement

The original contributions presented in the study are included in the article/**Supplementary Material**. Further inquiries can be directed to the corresponding author.

## Ethics statement

The studies involving human participants were reviewed and approved by the Medical Ethics Committee of Harbin Medical University. The patients/participants provided their written informed consent to participate in this study.

## Author contributions

HS and YLL contributed equally to this work. YPL had full access to all of the data in the initial study and take responsibility for the integrity of the data and the accuracy of the data analysis. YPL and YSZ contributed to study conception and design. YPL, HS, YW and YXZ contributed to DNA preparation and bisulfite modification. YPL, HRS, YBNW, and YLL contributed to MS-HRM. YPL, HS, YXZ, and YSZ contributed to collection and assembly of data. YSZ, YPL, HS, and YLL contributed to the analysis and interpretation of data. YPL, YLL, YXZ and YSZ contributed to sample collection. YPL, HS, YLL, YXZ and YSZ contributed to the drafting of the initial versions of the manuscript. All authors contributed to the article and approved the submitted version.YPL and YSZ were responsible for study supervision.
