# Supplementary material for: Insulin-like growth factor 2 hypermethylation in peripheral blood leukocytes and colorectal cancer risk and prognosis: a propensity score analysis
Source: Front Oncol. 2023 May 5;13:971435. doi: 10.3389/fonc.2023.971435 (PMC10198613; doi:10.3389/fonc.2023.971435)
Supplement: Supplementary file 1 [file DataSheet_1.pdf]

**Insulin-like growth factor 2 hypermethylation in peripheral blood leukocytes and colorectal cancer risk and prognosis: A propensity score analysis**

HongRu Sun, YanLong Liu, YuXue Zhang, Yibaina Wang, YaShuang Zhao, YuPeng Liu

# *Supplementary Material*

## 1. Supplementary Tables

**Supplementary Table 1.** Main characteristics of participants of the initial and validation studies in the CRC risk analysis.
**Supplementary Table 2.** Main characteristics of patients of the initial and validation cohorts in the CRC prognosis analysis.
**Supplementary Table 3.** Comparisons of participant characteristics and covariates between CRC cases and controls before and after propensity score adjustment in the initial case-control study in CRC risk analysis.
**Supplementary Table 4.** Comparisons of baseline characteristics of CRC patients before and after propensity score adjustment in the initial cohort in CRC prognosis analysis.
**Supplementary Table 5.** The Associations between PBL IGF2 methylation and the risk of developing CRC using propensity score based methods.
**Supplementary Table 6.** The Associations between PBL IGF2 methylation and CRC patient prognosis using PS-based methods.
**Supplementary Table 7.** E-values for PS-adjusted effect estimates.
**Supplementary Table 8.** The Associations between PBL IGF2 methylation and UICC stage, serum CEA level, WBC counts, and whether or not included in the survival analysis among CRC patients.
**Supplementary Table 9.** The Associations of PBL IGF2 methylation and CRC patient prognosis with or without WBC counts included in the PS model.

**Supplementary Table 1.** Main characteristics of participants of the initial and validation studies in the CRC risk analysis.
BMI, body mass index; CRC, colorectal cancer; NA, not available; SD, standard deviation.

| Characteristics | Initial study | | | Validation study | | | | | |
|---|---|---|---|---|---|---|---|---|---|
| | | | | GSE51032 | | | GSE89093 | | |
| | **No. of Cases** (%) | **No. of Controls** (%) | ***P*-value** | **No. of Cases** (%) | **No. of Controls** (%) | ***P*-value** | **No. of Cases** (%) | **No. of Controls** (%) | ***P*-value** |
| Total number | 428 | 428 | | 166 | 424 | | 12 | 46 | |
| Age (years), mean (SD) | 59.37 (10.30) | 59.36 (10.35) | 0.9892 | 55.08 (6.73) | 53.22 (7.19) | 0.0044 | 63.59 (6.11) | 60.94 (9.98) | 0.3857 |
| < 60 | 219 (51.17) | 217 (50.70) | 0.9517 | 128 (77.11) | 348 (82.08) | 0.1694 | 5 (41.67) | 22 (47.83) | 0.9553 |
| ≥ 60 | 209 (48.83) | 211 (49.30) | | 38 (22.89) | 76 (17.92) | | 7 (58.33) | 24 (52.17) | |
| Gender | | | | | | | | | |
| Male | 266 (62.15) | 266 (62.15) | 1.0000 | 87 (52.41) | 84 (19.81) | <0.0001 | - | - | NA |
| Female | 162 (37.85) | 162 (37.85) | | 79 (47.59) | 340 (80.19) | | 12 (100.00) | 46 (100.00) | |
| BMI (Kg/m2), mean (SD) | 23.38 (3.30) | 27.80 (9.56) | <0.0001 | NA | NA | | NA | NA | |
| < 24.00 | 247 (57.71) | 172 (40.18) | <0.0001 | NA | NA | | NA | NA | |
| ≥ 24.0 | 181 (42.29) | 256 (59.82) | | NA | NA | | NA | NA | |
| Tumour site | | | | | | | | | |
| Colon | 177 (41.35) | NA | NA | 154 (92.77) | NA | | 10 (83.33) | NA | |
| Rectum | 251 (58.65) | NA | NA | 12 (7.23) | NA | | 2 (16.67) | NA | |
| UICC/AJCC stage | | | | | | | | | |
| Stage I | 26 (6.07) | NA | NA | NA | NA | | NA | NA | |
| Stage II | 205 (47.90) | NA | NA | NA | NA | | NA | NA | |
| Stage III | 160 (37.38) | NA | NA | NA | NA | | NA | NA | |
| Stage IV | 15 (3.50) | NA | NA | NA | NA | | NA | NA | |
| Unknown | 22 (5.14) | NA | NA | NA | NA | | NA | NA | |

**Supplementary Table 2.** Main characteristics of patients of the initial and validation cohorts in the CRC prognosis analysis.

BMI, body mass index; CRC, colorectal cancer; NA, not available; SD, standard deviation.

| Characteristic | Initial Cohort Study | | Validation Cohort Study | |
|---|---|---|---|---|
| | **Patients with Blood Samples** | **Patients with Tissue Samples** | **EPIC-Italy CRC Cohort with Blood Samples** | **TCGA Dataset with Tissue Samples** |
| Total | 281 | 297 | 166 | 380 |
| Age (years), mean (SD) | 59.12 (10.11) | 58.89 (11.43) | 55.08 (6.73) | 64.34 (13.08) |
| < 60 | 146 (52.00) | 163 (54.88) | 128 (77.11) | 147 (38.68) |
| ≥ 60 | 135 (48.00) | 134 (45.12) | 38 (22.89) | 233 (61.32) |
| Sex | | | | |
| Male | 170 (60.50) | 172 (57.91) | 87 (52.41) | 208 (54.74) |
| Female | 111 (39.50) | 125 (42.09) | 79 (47.59) | 172 (45.26) |
| BMI (Kg/m2), mean (SD) | 23.57 (3.22) | 23.54 (3.45) | NA | NA |
| < 24.00 | 158 (56.23) | 173 (58.25) | NA | NA |
| ≥ 24.00 | 123 (43.77) | 124 (41.75) | NA | NA |
| Tumour location | | | | |
| Colon | 113 (40.20) | 98 (33.00) | 154 (92.77) | 286 (75.26) |
| Rectum | 168 (59.80) | 199 (67.00) | 12 (7.23) | 94 (24.74) |
| UICC/AJCC stage | | | | |
| Stage I | 29 (10.30) | 32 (10.77) | NA | 59 (15.53) |
| Stage II | 124 (44.10) | 131 (44.11) | NA | 143 (37.63) |
| Stage III | 114 (40.60) | 113 (38.05) | NA | 122 (32.11) |
| Stage IV | 14 (5.00) | 21 (7.07) | NA | 56 (14.74) |
| Pathological morphology | | | | |
| Protruding type | 178 (63.30) | 187 (62.96) | NA | NA |
| Ulcerative type | 103 (36.70) | 110 (37.04) | NA | NA |
| Tumour differentiation | | | | |
| Well to moderate | 235 (83.60) | 258 (86.87) | NA | NA |
| Poor | 46 (16.40) | 39 (13.13) | NA | NA |
| Histological classification | | | | |
| Adenocarcinoma or mucinous adenocarcinoma | 264 (94.00) | 281 (94.61) | NA | 334 (87.89) |
| Other types | 17 (6.00) | 16 (5.39) | NA | 46 (12.11) |
| History of polyps | | | | |
| No | NA | NA | NA | 292 (76.84) |
| Yes | NA | NA | NA | 88 (23.16) |

**Supplementary Table 3.** Comparisons of participant characteristics and covariates between CRC cases and controls before and after propensity score adjustment in the initial case-control study in CRC risk analysis.

BMI, body mass index; CRC, colorectal cancer; SD, standard deviation.

| **Characteristic** | **Overall** | **Case** | **Control** | **Standardised Difference** (%) | |
|---|---|---|---|---|---|
| | | | | **Before adjustment** | **After adjustment** |
| Propensity Score | | | | 115.12 | 0.24 |
| Age (years), mean (SD) | 59.36 (10.31) | 59.37 (10.30) | 59.36 (10.35) | 0.11 | 7.05 |
| < 60 | 436 (50.93) | 219 (51.17) | 217 (50.70) | -0.93 | 1.26 |
| ≥ 60 | 420 (49.07) | 209 (48.83) | 211 (49.30) | | |
| Sex | | | | | |
| Male | 532 (62.15) | 266 (62.15) | 266 (62.15) | 0.00 | -6.14 |
| Female | 324 (37.85) | 162 (37.85) | 162 (37.85) | | |
| BMI (Kg/m2), mean (SD) | 25.55 (7.37) | 23.38 (3.30) | 27.80 (9.56) | -134.25 | 9.43 |
| < 24.00 | 419 (48.95) | 247 (57.71) | 172 (40.18) | -35.43 | 3.27 |
| ≥ 24.00 | 437 (51.05) | 181 (42.29) | 256 (59.82) | | |
| Family history of any cancers other than CRC in first-degree relatives | | | | | |
| No | 726 (84.81) | 356 (83.18) | 370 (86.45) | 8.73 | 3.62 |
| Yes | 130 (15.19) | 72 (16.82) | 58 (13.55) | | |
| Smoking | | | | | |
| No | 460 (53.74) | 243 (56.78) | 217 (50.70) | -12.25 | 0.09 |
| Yes | 396 (46.26) | 185 (43.22) | 211 (49.30) | | |
| Occupational physical activity | | | | | |
| Blue-collar worker | 554 (64.72) | 229 (53.50) | 325 (75.93) | 44.92 | 8.43 |
| White-collar worker | 302 (35.28) | 199 (46.50) | 103 (24.07) | | |
| Barbecue food | | | | | |
| <1 time/week | 663 (77.45) | 324 (75.70) | 339 (79.21) | 8.16 | -0.31 |
| ≥ 1 time/week | 193 (22.55) | 104 (24.30) | 89 (20.79) | | |
| Coarse grains | | | | | |
| < 50 g/week | 343 (40.07) | 204 (47.66) | 139 (32.48) | -30.37 | -6.22 |
| ≥ 50 g/week | 513 (59.93) | 224 (52.34) | 289 (67.52) | | |
| Fish stewed with brown sauce | | | | | |
| < 1 time/week | 601 (70.21) | 274 (64.02) | 327 (76.40) | 25.77 | -7.06 |
| ≥ 1 time/week | 255 (29.79) | 154 (35.98) | 101 (23.60) | | |
| Fresh fruits | | | | | |
| < 2 times/week | 437 (51.05) | 217 (50.70) | 220 (51.40) | 1.40 | -5.23 |
| ≥ 2 times/week | 419 (48.95) | 211 (49.30) | 208 (48.60) | | |
| Fried food | | | | | |
| <1 time/month | 639 (74.65) | 295 (68.93) | 344 (80.37) | 24.71 | 3.46 |
| ≥ 1 time/month | 217 (25.35) | 133 (31.07) | 84 (19.63) | | |
| Green vegetables | | | | | |
| < 100 g/day | 785 (91.71) | 385 (89.95) | 400 (93.46) | 11.64 | -5.18 |
| ≥ 100 g/day | 71 (8.29) | 43 (10.05) | 28 (6.54) | | |
| Leftover | | | | | |
| < 1 time/week | 534 (62.38) | 252 (58.88) | 282 (65.89) | 14.23 | -6.62 |
| ≥ 1 time/week | 322 (37.62) | 176 (41.12) | 146 (34.11) | | |
| Pork | | | | | |
| < 250 g/week | 472 (55.14) | 221 (51.64) | 251 (58.64) | 14.01 | -6.35 |
| ≥ 250 g/week | 384 (44.86) | 207 (48.36) | 177 (41.36) | | |

**Supplementary Table 4.** Comparisons of baseline characteristics of CRC patients before and after propensity score adjustment in the initial cohort in CRC prognosis analysis.

BMI, body mass index; CA19-9, carbohydrate antigen 19-9; CEA, carcinoembryonic antigen; CRC, colorectal cancer; IQR, inter-quartile range; PBLs, peripheral blood leukocytes; SD, standard deviation; WBC, white blood cell.

| Characteristic | Overall | IGF2 Methylation in PBLs | | Standardised Difference (%) | |
|---|---|---|---|---|---|
| | | Hypomethylation | Hypermethylation | Before adjustment | After adjustment |
| Propensity Score | | | | 100.26 | 0.93 |
| Age (years), mean (SD) | 59.12 (10.11) | 58.52 (10.15) | 60.79 (9.88) | 22.95 | -6.57 |
| < 60 | 146 (52.00) | 115 (55.80) | 31 (41.30) | -29.23 | 11.48 |
| ≥ 60 | 135 (48.00) | 91 (44.20) | 44 (58.70) | | |
| Sex | | | | | |
| Male | 170 (60.50) | 128 (62.10) | 42 (56.00) | 12.28 | -4.16 |
| Female | 111 (39.50) | 78 (37.90) | 33 (44.00) | | |
| BMI (Kg/m2), mean (SD) | 23.57 (3.22) | 23.88 (3.26) | 22.70 (2.95) | -40.02 | -3.17 |
| < 24.00 | 158 (56.23) | 105 (50.97) | 53 (70.67) | 42.97 | -1.77 |
| ≥ 24.00 | 123 (43.77) | 101 (49.03) | 22 (29.33) | | |
| Family history of any cancers other than CRC in first-degree relatives | | | | | |
| No | 231 (82.20) | 168 (81.60) | 63 (84.00) | 6.63 | 1.49 |
| Yes | 50 (17.80) | 38 (18.40) | 12 (16.00) | | |
| Smoking | | | | | |
| No | 171 (60.90) | 132 (64.10) | 39 (52.00) | -24.01 | -2.57 |
| Yes | 110 (39.10) | 74 (35.90) | 36 (48.00) | | |
| Alcohol drinking | | | | | |
| No | 171 (60.90) | 123 (59.70) | 48 (64.00) | 8.88 | -0.74 |
| Yes | 110 (39.10) | 83 (40.30) | 27 (36.00) | | |
| Occupational physical activity | | | | | |
| Blue-collar worker | 136 (48.40) | 97 (47.10) | 39 (52.00) | 7.11 | 3.68 |
| White-collar worker | 145 (51.60) | 109 (52.90) | 36 (48.00) | | |
| Coarse grains | | | | | |
| < 50 g/week | 123 (43.80) | 83 (40.30) | 40 (53.30) | 25.97 | -2.26 |
| ≥ 50 g/week | 158 (56.20) | 123 (59.70) | 35 (46.70) | | |
| Fish stewed with brown sauce | | | | | |
| < 1 time/week | 190 (67.60) | 134 (65.00) | 56 (74.70) | 21.97 | 4.87 |
| ≥ 1 time/week | 91 (32.40) | 72 (35.00) | 19 (25.30) | | |
| Fried food | | | | | |
| <1 time/month | 190 (67.60) | 138 (67.00) | 52 (69.30) | 5.05 | 4.30 |
| ≥1 time/month | 91 (32.40) | 68 (33.00) | 23 (30.70) | | |
| Leftover | | | | | |
| < 1 time/week | 164 (58.40) | 119 (57.80) | 45 (60.00) | 4.53 | 5.34 |
| ≥ 1 time/week | 117 (41.60) | 87 (42.20) | 30 (40.00) | | |
| Pork | | | | | |
| < 250 g/week | 149 (53.00) | 103 (50.00) | 46 (61.30) | 21.14 | 3.02 |
| ≥ 250 g/week | 132 (47.00) | 103 (50.00) | 29 (38.70) | | |
| Tumour location | | | | | |
| Proximal colon (cecum to transverse) | 52 (18.50) | 37 (18.0) | 15 (20.0) | -5.06 | -7.25 |
| Distal colon (splenic flexure to sigmoid) | 61 (21.70) | 46 (22.3) | 15 (20.0) | -5.79 | -2.35 |
| Rectum | 168 (59.80) | 123 (59.7) | 45 (60.0) | 0.59 | 3.49 |

**Supplementary Table 4.** Continued

| | | | | | |
|---|---|---|---|---|---|
| Tumour size (mm), median (IQR) | 60 (27-150) | 60 (27-173) | 45 (24-125) | -60.67 | 1.97 |
| Preoperative CEA level (ng/mL), median (IQR) | 7.90 (2.40-19.65) | 9.15 (2.78-30.10) | 4.00 (2.10-15.20) | -3.47 | 0.44 |
| Preoperative CA19-9 level (U/mL), median (IQR) | 20.00 (9.90-39.32) | 20.87 (10.46-50.51) | 16.93 (8.27-32.96) | -9.45 | 1.86 |
| T stage (depth of tumour invasion) | | | | | |
| T1 or T2 | 38 (13.50) | 29 (14.10) | 9 (12.00) | 6.41 | 2.47 |
| T3 | 104 (37.00) | 77 (37.40) | 27 (36.00) | -2.85 | 4.27 |
| T4 | 139 (49.50) | 100 (48.50) | 39 (52.00) | 6.87 | 4.91 |
| N stage (number of positive lymph nodes) | | | | | |
| N0 | 155 (55.20) | 114 (55.30) | 41 (54.70) | -1.34 | 0.48 |
| N1 or N2 | 126 (44.80) | 92 (44.70) | 34 (45.30) | | |
| M stage (status of distant metastasis) | | | | | |
| M0 | 267 (95.00) | 193 (93.70) | 74 (98.70) | 43.11 | -6.10 |
| M1 | 14 (5.00) | 13 (6.30) | 1 (1.30) | | |
| UICC/AJCC stage | | | | | |
| I | 29 (10.30) | 23 (11.20) | 6 (8.00) | 11.62 | 6.83 |
| II | 124 (44.10) | 89 (43.20) | 35 (46.70) | 6.94 | 6.29 |
| III | 114 (40.60) | 81 (39.30) | 33 (44.00) | 9.36 | -2.10 |
| IV | 14 (5.00) | 13 (6.30) | 1 (1.30) | 0.59 | 0.69 |
| Pathological morphology | | | | | |
| Protruding type | 178 (63.30) | 135 (65.5) | 43 (57.3) | -21.02 | 3.07 |
| Ulcerative type | 103 (36.70) | 71 (34.5) | 32 (42.7) | | |
| Tumour differentiation | | | | | |
| Well to moderate | 235 (83.60) | 170 (82.50) | 65 (86.70) | 10.61 | 12.38 |
| Poor | 46 (16.40) | 36 (17.50) | 10 (13.30) | | |
| Histological classification | | | | | |
| Adenocarcinoma | 264 (94.00) | 194 (94.20) | 70 (93.30) | -3.35 | -3.12 |
| Other types | 17 (6.00) | 12 (5.80) | 5 (6.70) | | |
| Extent of lymph node dissection | | | | | |
| D1 or D2 or D3 | 171 (60.90) | 128 (62.10) | 43 (57.30) | -9.65 | 2.00 |
| D4 | 110 (39.10) | 78 (37.90) | 32 (42.70) | | |
| Postoperative adjuvant chemotherapy | | | | | |
| No | 153 (54.40) | 108 (52.40) | 45 (60.00) | 15.35 | -1.33 |
| Yes | 128 (45.60) | 98 (47.60) | 30 (40.00) | | |
| Postoperative adjuvant radiotherapy | | | | | |
| No | 259 (92.20) | 191 (92.70) | 68 (90.70) | -5.28 | 10.43 |
| Yes | 22 (7.80) | 15 (7.30) | 7 (9.30) | | |
| WBC counts, median (IQR) | 6.50 (5.50-7.75) | 6.40 (5.48-7.90) | 6.50 (5.60-7.40) | 8.12 | 1.32 |
| Lymphocytes percentage | 26.90 (20.80-33.30) | 27.00 (20.75-32.20) | 26.50 (21.30-35.00) | 13.50 | -3.45 |
| Neutrophil percentage | 63.80 (55.55-70.45) | 64.30 (56.48-70.40) | 62.30 (54.60-70.80) | 11.51 | -5.34 |
| Monocyte percentage | 6.18 (4.10-7.91) | 6.14 (4.04-7.89) | 6.36 (4.10-8.00) | -56.11 | 11.87 |
| Eosinophils percentage | 0.70 (0.30-1.76) | 0.70 (0.30-1.73) | 0.80 (0.40-1.78) | 5.50 | 7.92 |
| Basophil percentage | 0.10 (0.00-0.65) | 0.15 (0.00-0.66) | 0.10 (0.00-0.60) | -15.64 | -1.32 |

**Supplementary Table 5.** Sensitivity analyses using additional propensity score based methods for CRC risk analysis in the initial case-control study.

*Test for heterogeneity between ORs was conducted by using random effect models with Comprehensive Meta Analysis (version 2.2.046).

CI, confidence interval; CRC, colorectal cancer; OR, odds ratio; PBL, peripheral blood leukocyte; PS, propensity score; IPTW, inverse probability of treatment weights with propensity score.

| Effect Estimates | OR (95% CI) | P-value | P-value for difference between methods* |
|---|---|---|---|
| Univariate | 2.8172 (1.8799-4.1917) | <0.00001 | 0.9921 |
| PS-adjusted | 2.5893 (1.6584-4.0429) | <0.00001 | |
| PS-stratified | 2.5526 (1.6363-3.9820) | <0.00001 | |
| IPTW | 2.5994 (1.6745-4.0350) | <0.00001 | |
| PS-matching | 2.4286 (1.6383-3.6000) | <0.00001 | |

**Supplementary Table 6.** Sensitivity analyses using additional propensity score based methods for CRC survival analysis in the initial CRC cohort.

*Test for heterogeneity between ORs was conducted by using random effect models with Comprehensive Meta Analysis (version 2.2.046).

CI, confidence interval; CRC, colorectal cancer; CSS, cancer specific survival; DFS, disease free survival; HR, hazard ratio; OS, overall survival; PBL, peripheral blood leukocyte; PS, propensity score.

| Outcome | Univariate HR, 95% CI | P-value | PS-adjusted HR, 95% CI | P-value | PS-stratified HR, 95% CI | P-value | IPTW-HR, 95% CI | P-value | PS-matched HR, 95% CI | P-value | P-value for difference between methods* |
|---|---|---|---|---|---|---|---|---|---|---|---|
| OS | 0.5477 (0.3550-0.8450) | 0.0065 | 0.4701 (0.2918-0.7573) | 0.0019 | 0.4485 (0.2808-0.7162) | 0.0008 | 0.4834 (0.3016-0.7748) | 0.0026 | 0.4481 (0.2805-0.7160) | 0.0008 | 0.9707 |
| CSS | 0.5576 (0.3579-0.8687) | 0.0098 | 0.4901 (0.3010-0.7981) | 0.0042 | 0.4727 (0.2292-0.7647) | 0.0023 | 0.4954 (0.3052-0.8042) | 0.0046 | 0.4614 (0.2850-0.7470) | 0.0017 | 0.9824 |
| RFS | 0.5989 (0.3855-0.9307) | 0.0226 | 0.5263 (0.3249-0.8525) | 0.0091 | 0.5218 (0.3246-0.8388) | 0.0072 | 0.4915 (0.2927-0.8254) | 0.0074 | 0.5782 (0.3649-0.9160) | 0.0196 | 0.9787 |

**Supplementary Table 7.** E-values for PS-adjusted effect estimates.

CI, confidence interval; CRC, colorectal cancer; CSS, cancer specific survival; DFS, disease free survival; HR, hazard ratio; OS, overall survival; PBL, peripheral blood leukocyte; PS, propensity score.

| **Items** | **Effect Estimates, 95% CI** | **E-value for Point Estimate** | **E-value for Lower CI Limit** |
|---|---|---|---|
| **PBL IGF2 methylation and CRC incidence risk** | | | |
| **Case-control study** | **PS-adjusted OR, 95% CI** | | |
| Initial study | 2.5737 (1.6445-4.0278) | 4.5862 | 2.6740 |
| Validation study | | | |
| GSE51032 | 2.2122 (1.2845, 3.8098) | 3.8498 | 1.8890 |
| GSE89093 | 10.652 (1.2649, 89.706) | 20.7917 | 1.8438 |
| Pooled studies | 2.5146 (1.7877, 3.5372) | 4.4662 | 2.9744 |
| **PBL IGF2 methylation and CRC patient prognosis** | | | |
| **Cohort study** | **PS-adjusted HR, 95% CI** | | |
| OS | 0.4701 (0.2918-0.7573) | 2.7524 | 1.7196 |
| CSS | 0.4901 (0.3010-0.7981) | 2.6532 | 1.6137 |
| DFS | 0.5263 (0.3249-0.8525) | 2.4885 | 1.4783 |

**Supplementary Table 8.** The Associations between PBL IGF2 methylation status and UICC stage, serum CEA level, WBC counts, and whether or not included in the survival analysis among CRC patients.

CEA, carcinoembryonic antigen; CRC, colorectal cancer; IQR, inter-quartile range; PBL, peripheral blood leukocyte; WBC, white blood cell.

| **Factors** | **Total** | **IGF2 methylation in PBLs** | | **Pearson Chi-Square** | **P-value** |
|---|---|---|---|---|---|
| | | **Hypomethylation** | **Hypermethylation** | | |
| UICC stage | | | | 4.2992 | 0.2309 |
| I + II | 231 | 184 | 47 | | |
| III | 160 | 118 | 42 | | |
| IV | 15 | 14 | 1 | | |
| Unkown | 22 | 18 | 4 | | |
| CEA levels (ng/mL) | | | | 6.2150 | **0.0127** |
| Lower (≤ 5 ng/mL) | 123 | 81 | 42 | | |
| Higher (> 5 ng/mL) | 158 | 125 | 33 | | |
| Included in the survival analysis | | | | 0.6524 | 0.4193 |
| Included | 281 | 216 | 75 | | |
| Excluded | 147 | 118 | 29 | | |
| | | | | **Mann-Whitney U** | **P-value** |
| WBC counts, median (IQR) | 6.50 (5.50-7.75) | 6.40 (5.48-7.90) | 6.50 (5.60-7.40) | 7761.5 | 0.9520 |
| Lymphocytes percentage | 26.90 (20.80-33.30) | 27.00 (20.75-32.20) | 26.50 (21.30-35.00) | 8258.0 | 0.3760 |
| Neutrophil percentage | 63.80 (55.55-70.45) | 64.30 (56.48-70.40) | 62.30 (54.60-70.80) | 7509.5 | 0.7210 |
| Monocyte percentage | 6.18 (4.10-7.91) | 6.14 (4.04-7.89) | 6.36 (4.10-8.00) | 7999.0 | 0.6490 |
| Eosinophils percentage | 0.70 (0.30-1.76) | 0.70 (0.30-1.73) | 0.80 (0.40-1.78) | 8242.5 | 0.3900 |
| Basophil percentage | 0.10 (0.00-0.65) | 0.15 (0.00-0.66) | 0.10 (0.00-0.60) | 7436.5 | 0.6260 |

**Supplementary Table 9.** The Associations of PBL IGF2 methylation and CRC patient prognosis with or without WBC counts included in PS models.
CI, confidence interval; CRC, colorectal cancer; CSS, cancer specific survival; DFS, disease free survival; HR, hazard ratio; OS, overall survival; PBL, peripheral blood leukocyte; PS, propensity score; WBC, white blood cell.

| **Outcome** | **Included WBC count in the PS-model** | **PS-adjusted HR, 95% CI** | **P-value for difference between models** |
|---|---|---|---|
| OS | Yes | 0.4701 (0.2918-0.7573) | 0.9281 |
| | No | 0.4847 (0.3051-0.7700) | |
| CSS | Yes | 0.4901 (0.3010-0.7981) | 0.9208 |
| | No | 0.5076 (0.3159-0.8157) | |
| DFS | Yes | 0.5263 (0.3249-0.8525) | 0.8537 |
| | No | 0.5607 (0.3506-0.8966) | |
